# Biotoxicity of essential oil of *Eucalyptus citriodora* Hook (Myrtaceae) to dust mites

**DOI:** 10.3389/fpls.2025.1708798

**Published:** 2025-12-04

**Authors:** Haiming Cai, Pusong Xie, Xu Zhang, Zhibin Lin, Zhimin Xu, Shuting Chen, Peiyao Ruan, Komivi S. Akutse, Shanshan Li, Huiquan Lin, Ziyi Wu, Yongwen Lin

**Affiliations:** 1 Zhangzhou Affiliated Hospital of Fujian Medical University, Zhangzhou, Fujian, China; 2 International Centre of Insect Physiology and Ecology, Nairobi, Kenya; 3 Unit of Environmental Sciences and Management, North-West University, Potchefstroom, South Africa; 4 Fujian Academy of Agricultural Sciences Institute of Subtropical Agriculture, Zhangzhou Institute of Technology, Zhangzhou, Fujian, China; 5 College of Landscape Architecture and Art, Fujian Agriculture and Forestry University, Fujian, China; 6 Fujian Zhanglong Group Co., Ltd., Fujian, China; 7 College of Food Engineering, Zhangzhou Institute of Technology, Zhangzhou, Fujian, China

**Keywords:** citronellol, *Dermatophagoides pteronyssinus*, *Dermatophagoides farinae*, toxicity, repellency, environmental control

## Abstract

**Introduction:**

Dust mites are a prevalent indoor allergen contributing to respiratory diseases like allergic rhinitis and asthma. *Eucalyptus citriodora* essential oil, known for its balsamic odor and repellent effects on various pests, has been scantily investigated for its impacts on dust mites.

**Methods:**

The chemical composition of the essential oil and its head-space extracted from *E. citriodora* was determined using gas chromatography-mass spectrometry (GC-MS). The toxicity of the oil and its compounds were assessed through contact-fumigant and vapor-phase mortality bioassays. Repellent effects were evaluated using a fabric-contact assay. Data were analyzed using probit regression to determine LC_50_ values.

**Results:**

The essential oil contained seven main compounds, and citronellal, citronellol and citronellyl acetate were the most abundant in the oil's volatile, accounting fora total of 88.22%. Citronellal exhibited the highest toxicity, and the essential oil itself showed strong toxicity with the LC_50_ of 63.94 and 84.53 μL/cm against *Dermatophagoides pteronyssinus*, 60.72 and 75.88 μL/cm against *Dermatophagoides farinae*, respectively. In vapor-phase assays, citronellal and ethyl phenylacetate caused 100% mortality.

**Discussion:**

Citronellol had the highest repellent effect, and the essential oil, 1,8-cineole, and citronellyl acetate also showed significant repellency rates. *E. citriodora* essential oil and its compounds particularly citronellal and citronellol, showed high potential for effective dust mite control due to their natural origin, strong toxicity and repellency impacts. Thus, *E. citriodora* essential oil is a natural, eco-friendly alternative to synthetic acaricides, providing a scientific basis for the control of indoor dust mite allergies.

## 1 Introduction

Dust mites (*Dermatophagoides pteronyssinus* and *Dermatophagoides farinae*) are a common indoor allergen, widely present in environments where humans live and work, such as homes, schools, and offices (Aggarwal and Senthilkumaran, 2024; Caraballo, 2024; Klain et al., 2024). They feed on human dander, proliferate rapidly, and release allergenic proteins such as Der p1 and Der p2 that are contained in fecal particles and carcass remains (Aggarwal and Senthilkumaran, 2024). The allergenic proteins that induce allergic reaction are significant contributors to respiratory diseases such as allergic rhinitis and asthma (Marko and Pawliczak, 2023; Rodriguez-Plata et al., 2023; Zheng et al., 2023; Turki et al., 2024). Dust mites thrive in warm, humid environments, and the adult stage is most harmful for spreading respiratory diseases due to its high production of allergenic feces and debris that become airborne and inhaled (Marko and Pawliczak, 2023; Zheng et al., 2023; Turki et al., 2024). In recent years, with the improvement of people’s living standards and living environments, the demand for dust mite control has significantly increased.

Considerable research has been conducted on dust mite control using chemicals and natural products (Maruoka et al., 2020; Gómez-Gutiérrez et al., 2023; Espinosa-Zaragoza et al., 2024). For chemical agents, some branched chain fatty acids (2-ethylhexanoic acid, 2-butyloctanoic acid, and isopalmitic acid) were found to be toxic to more than 50% dust mites in 90 min (Maruoka et al., 2020). Chemicals are also used to enhance the acaricidal activity of biological control agents, such as microbial strains or natural products against dust mites (Hussain and AlJabr, 2020). For natural products, three *Serratia* strains, *S. ureilytica* UTS2, UTS3, and UTS4 isolated from *Mimosa pudica* were found to hold acaricidal activity for controlling dust mite *in vitro* (Espinosa-Zaragoza et al., 2024). However, chemical methods may pose high risks to human health and the environment, while biological approaches are difficult to implement in household environments. Plant-derived essential oils have gained significant attention in pest management due to their natural origin, safety, environmental friendliness, and lack of harmful residues. It was found that cinnamon essential oil, hiba essential oil, and orange essential oil exhibited a strong acaricidal effect against house dust mites (Kim et al., 2015; Jung et al., 2020; Sabahi et al., 2020).

*Eucalyptus citriodora* Hook. (Myrtaceae), a medium-sized evergreen tree native to northeastern Australia with a slender trunk, lance-shaped glossy leaves (emitting a lemon scent when crushed), and resilience to drought and pollution, has long been used in folk medicine, which essential oil serves as an anti-inflammatory, antiseptic, and expectorant for respiratory issues. *E. citriodora* essential oil has been reported to have potential application as a natural pesticide against some key pests (El-Sakhawy et al., 2023). *E. citriodora* essential oil also showed antimicrobial potential against *Staphylococcus aureus*, *Candida albicans* and *Hemileia vastatrix* (Pinheiro et al., 2020; Caetano et al., 2022; Fayez et al., 2023). However, research on the repellent effect of *E. citriodora* essential oil on dust mites is relatively scarce, and existing studies lack depth and clarity on these aspects. Therefore, this study aims to further explore the repellent effect of *E. citriodora* essential oil on dust mites, providing a scientific basis for the development of novel, environmentally friendly dust mite control methods. The primary objective of this study is to (1) clarify the dose-response relationship of *E. citriodora* essential oil in repelling dust mites through bioassay experiments, (2) determine the chemical components and head-space of *E. citriodora* essential oil using Gas chromatography-mass spectrometry (GC-MS), and (3) assess the repellent effect and mechanisms of *E. citriodora* essential oil on dust mites.

## 2 Materials and methods

### 2.1 Mite colony and plant product

*Dermatophagoides pteronyssinus* and *D. farinae* were harvested using vacuum sampling from mattresses, carpets, and upholstered furniture in a residential bedroom in Zhangzhou, city (117.633919°E, 24.521705° N, China) and subsequently maintained for a period of six months without exposure to any known acaricides. Mite manipulation was performed under a dissecting microscope (×20–40 magnification) to ensure precision: adult mites (7–10 days old) were gently transferred using a fine brush (0.5 mm bristle diameter) to avoid physical damage. Mites with fine, fingerprint-like cuticular striations covering their body surfaces and a semi-transparent body are *D. farinae*, while mites with relatively sparse striations arranged in a wavy or longitudinal pattern and a milky yellow body color are *D. pteronyssinus.* These mites were reared within Petri dishes measuring 8.5 cm in diameter and 5.5 cm in depth, which were filled with a sterilized substrate composed of an equal parts mixture by weight of wheat bran and dried yeast. Previous studies demonstrated the physics of dust mites were regulated by the temperature and humidity obviously (Colloff, 1987; Vackova et al., 2023). The mites were kept in the incubator, maintaining at a temperature of 25 ± 1 °C and a relative humidity of 75%, under constant darkness. The dried yeast component of the diet was procured from Angel Yeast Co., Ltd., based in Yichang, China.

Essential oil of *Eucalyptus citriodora* Hook was purchased from Xiya Reagent, Shangdong, China. The seven artificial chemicals used in the study were listed in **Table 1**. For the quantitative structure-activity relationship (QSAR) analysis, the molecular weight (MW), vapor pressure (VP), and steric parameters of the test compounds were derived using ACD/Boiling Point and Vapor Pressure software (ACD/Labs Online, provided by Advanced Chemistry Development, Inc., Montreal, Canada). The calculated values are summarized in **Table 1**. All chemical reagents employed in the study were of analytical grade and were sourced from Xilong Scientific Co., Ltd., Shangxi, China.

**Table 1:** The seven main compounds examined in this study.

Chemicals	Molecular weight	Vapor pressure (hPa at 25°C)
1,8-Cineole	154.25	1.6
Citronellol	156.27	0.02
Citronellal	154.25	0.85
(-)-Globulol	222.37	0.00017
Ethyl phenylacetate	164.2	0.1-0.5
Citronellyl acetate	198.3	0.0137
β-Caryophyllene	204.35	0.045

### 2.2 Chromatographic analysis of the essential oil

Gas chromatography-mass spectrometry (GC-MS) analysis was conducted using a PerkinElmer Clarus 680 T gas chromatograph-mass spectrometer (Fort Belvoir, VA). The separation was achieved on an Agilent DB-5MS capillary column (30 m length, 0.25 mm internal diameter, Folsom, CA). The initial oven temperature was set at 50°C for 5 minutes, followed by a programmed ramp to 280°C at a rate of 5°C per minute, concluding with a 10-minute isothermal hold at 280°C. The helium carrier gas flow rate was maintained at 1.0 mL/min, mode split (50:1), injection volume was 1 μL. The ion source was operated at 250°C, while the interface temperature was set to 260°C. Electron ionization mass spectra were acquired at 70 eV, and the sector mass analyzer scanned a mass-to-charge (m/z) range from 35 to 550 every 0.2 seconds. Identification of the chemical constituents was achieved through spectral comparison with reference standards contained within a mass spectral library.

The volatile fraction of the essential oil was analyzed using a head-space solid-phase microextraction (HS-SPME) method coupled with GC-MS to isolate and identify volatile compounds. For volatile analysis, 1 g of the essential oil was placed in a 20 mL headspace vial, equilibrated at 60°C for 30 min, and then sampled using a 50 μm polydimethylsiloxane fiber (Supelco, Bellefonte, PA, USA) for 40 min. The fiber was desorbed in the GC inlet at 250°C for 5 min. The GC-MS conditions were identical to those described for the essential oil analysis.

### 2.3 Contact-fumigant mortality bioassay

A fabric-contact fumigant mortality bioassay was conducted to assess the biotoxicity of *E. citriodora* oil and seven selected compounds against adult dust mites, specifically *D. pteronyssinus* and *D. farinae*. Following preliminary testing, five concentrations of each test substance were prepared in 50 μL of ethanol and applied to black cotton fabric disks with a diameter of 5 cm. After application, the fabric disks were air-dried in a fume hood for one minute. Each disk was then positioned at the base of a disposable Petri dish (5 cm diameter × 1 cm height), and groups of 30 adult mites (both sexes, aged 7–10 days) were introduced onto the treated fabric. The Petri dishes were sealed with their original lids and further secured with Bemis Parafilm M (Neenah, WI). Control treatments were performed using disks treated with 50 μL of ethanol alone.

Both treated and control mites (exposed to ethanol only) were maintained under identical conditions consistent with those used for colony maintenance as described above. Mortality assessments were conducted 24 hours post-exposure using a dissecting microscope at a magnification of ×20. A mite was deemed dead if it failed to exhibit any movement in its body or appendages when stimulated with a fine wooden probe, as outlined in previous protocols (Kim et al., 2015). To account for the fact that all bioassays could not be executed simultaneously, treatments were organized into temporal blocks to control for time-dependent variability, each including a respective control group. For each block, freshly prepared solutions were utilized, and all treatments were replicated three times, with each replicate consisting of 30 adult mites.

### 2.4 Vapor-phase mortality bioassay

To ascertain whether the lethality of *E. citriodora* essential oil and the seven selected components against adult *D. pteronyssinus* and *D. farinae* was due to contact or fumigant action, a closed versus open container treatment protocol was employed. Groups of 30 adult mites (both sexes, aged 7–10 days) were placed on untreated cotton fabric disks positioned at the base of polystyrene containers (5 cm diameter × 2 cm height). Each container was sealed with a tight-fitting lid equipped with a fine wire mesh covering a central 4.5 cm diameter aperture. Approximately double concentration of the contact-fumigant LC_50_ values of each test material were applied to 5 cm diameter filter papers. These treated filter papers were then placed over the wire mesh, preventing direct contact between the mites and the test substances. Containers were sealed either with a solid lid (closed container treatment) or a lid with a 4.8 cm diameter central opening (open container treatment) to evaluate the vapor-phase toxicity of the substances. Control filter papers were treated with 50 μL of ethanol only. Mortality rates were assessed 24 hours after treatment, as previously detailed above. All bioassays were replicated three times, with each replicate containing 30 adult mites.

### 2.5 Repellent bioassay

**Figure 1:**
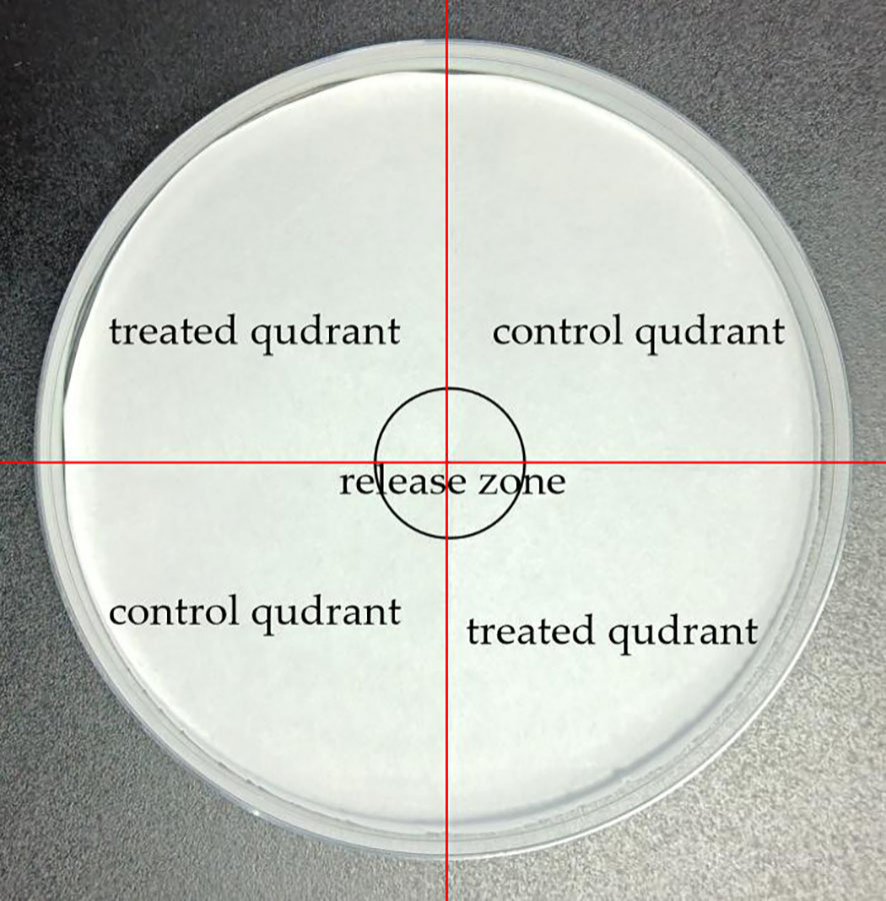
Petri dish set up for repellent bioassay.


For this assay, the LC_50_ concentration of the essential oil or chemicals effective against both dust mite species, *D. pteronyssinus* and *D. farinae*, was used. For each candidate chemical, a circular filter paper was divided into four equal quadrants with a central release zone, where two opposite quadrants were treated with 100 μL of test compounds and the other two served as 100 μL of solvent controls (**Figure 1**); mites were counted manually under a dissecting microscope by a trained observer at specified time points, with treatment labels blinded to minimize bias. A growth medium was placed on the filter paper for 5 minutes to allow its components to equilibrate with the test surface and maintain a stable microenvironment mimicking dust mite habitats, before being removed to isolate the treated area for behavioral observation. Subsequently, 30 adult mites were gently transferred using a brush to the compound-treated area of the cloth to avoid damage. Ethanol alone served as the control. Repellency was calculated using the following formula.

$$\text{Repellency}(\%)=1-\frac{\text{Number of mites in treated quadrant}}{\text{Total mites in treated and control quadrant}}$$

Ethanol alone served as the control. Both treated and control mites were maintained at 25 ± 1°C and 75% relative humidity (RH) in darkness. Instances where mites moved away from the treated area were recorded as avoidance behaviors. Repellency rates were evaluated after three hours. All treatments were replicated six times to ensure reliability and reproducibility of the results.

### 2.6 Data analysis

Mortality rates in the control groups were adjusted using Abbott’s formula. Concentration-mortality data were analyzed via probit regression to determine the lethal concentration for 50% of the tested subjects (LC_50_) for each experimental group. LC_50_ values were compared using non-overlapping 95% confidence intervals (CIs) to assess significant differences between compounds and mite species. For repellency data, mean repellency rates were calculated as described in Section 2.5, and mortality percentages and repellent rates were transformed using the arcsine square root transformation to stabilize variances and normalize the distribution prior to analysis of variance (ANOVA). Significant differences in repellency among treatments were identified using the Bonferroni multiple-comparison procedure, while a t-test was employed to evaluate differences between specific treatment pairs. Results are reported as means ± standard deviation (SD) based on the original, untransformed data.

## 3 Results

### 3.1 Main compounds of *Eucalyptus citriodora* essential oil

According to the GC-MS analysis, seven compounds (1,8-cineole, citronellol, citronellal, (-)-globulol, citronellyl acetate, β-caryophyllene, and ethyl phenylacetate) were identified in *E. citriodora* essential oil at the concentrations exceeding three percent (**Table 2**). Among these, three compounds (citronellol, citronellal, and citronellyl acetate) were present at different concentration levels greater than 10 percent in the volatile fraction of *E. citriodora* oil (**Table 2**). These seven compounds were designated as the primary constituents of both *E. citriodora* essential oil and its volatile components.

**Table 2:** Main compounds in the volatile of *Eucalyptus citriodora* essential oil.

Chemicals	(Mean value of peak area)%		
	Compounds in oil	Compounds involatile	Retention indices
1,8-Cineole	27.76 ± 2.12	0	1035
Citronellol	18.99 ± 1.55	15.89 ± 1.02	1242
Citronellal	6.89 ± 0.9	57 ± 3.23	1176
(-)-Globulol	5.95 ± 1.02	0	1262
Citronellyl acetate	3.67 ± 0.33	15.33 ± 0.98	1583
β-Caryophyllene	3.58 ± 0.32	0	1415
Ethyl phenylacetate	3.02 ± 0.54	0	1055

### 3.2 Contact-fumigant toxicity to dust mites

The toxicity data of *E. citriodora* essential oil and its seven main compounds against the two species of dust mites are presented in **Tables 3** and **4**. LC_50_ values were determined to assess the toxicity levels, where a lower value indicates higher toxicity. For *D. pteronyssinus*, citronellal exhibited the lowest LC_50_ value (63.94 μL/cm²) among all tested substances, while ethyl phenylacetate, 1,8-cineole, essential oil, and citronellyl acetate had LC_50_ values below 100 μL/cm². In contrast, β-caryophyllene, (-)-globulol, and citronellol demonstrated lower toxicity with LC_50_ values exceeding 100 μL/cm² (**Table 3**). For *D farinae*, citronellal, 1,8-cineole, ethyl phenylacetate, and essential oil showed higher toxicity with LC_50_ values ranging between 60.72-75.88 μL/cm²; whereas citronellyl acetate, citronellol, (-)-globulol, and β-caryophyllene displayed lower toxicity with LC_50_ values ranging between 104.08-208.77 μL/cm² (**Table 4**). Mortality rates for control treatments using only ethanol remained below 2%, which was used to adjust mortality rates in experimental treatments.

**Table 3:** Toxicity of *Eucalyptus citriodora* oil and the seven main compounds to *Dermatophagoides pteronyssinus* using a contact-fumigant mortality bioassay.

Compounds	LC50 (μL/cm^2^) (95%CL)	Slope ± SD	χ2	R^2^
Essential oil	84.53 (56.48~167.9)b	0.59 ± 0.11	19.53	0.83
1,8-Cineole	76.41 (53.25~135.21)b	0.65 ± 0.1	18.93	0.88
Citronellol	114.03 (96.39~139.55)ab	0.44 ± 0.03	5.335	0.89
Citronellal	63.94 (37.85~205.42)b	0.78 ± 0.19	35.83	0.80
(-)-Globulol	151.79 (131.96~178.57)a	0.33 ± 0.02	3.289	0.92
Ethyl phenylacetate	72.82 (48.54~145.82)b	0.69 ± 0.12	22.86	0.79
Citronellyl acetate	95.82 (72.87~139.94)b	0.52 ± 0.06	10.94	0.88
β-Caryophyllene	204.83 (177.18~242.84)a	0.24 ± 0.01	2.536	0.78

LC_50_ values with non-overlapping 95% confidence intervals (CIs) indicate significant differences between compounds (P< 0.05). Different letters follow LC_50_ value mean significantly different.

**Table 4:** Toxicity of *Eucalyptus citriodora* oil and the seven main compounds to *Dermatophagoides farinae* using a contact-fumigant mortality bioassay.

Compounds	LC_50_ (μL/cm^2^) (95%CL)	Slope ± SD	χ^2^	R^2^
Essential oil	75.88 (51.13~147.06)b	0.66 ± 0.11	21.22	0.78
1,8-Cineole	71.13 (47.94~137.74)b	0.7 ± 0.12	22.61	0.88
Citronellol	113.15 (94.07~141.88)b	0.44 ± 0.03	5.956	0.85
Citronellal	60.72 (33.53~319.69)b	0.82 ± 0.24	44.37	0.83
(-)-Globulol	141.04 (117.07~177.37)ab	0.35 ± 0.03	4.831	0.92
Ethyl phenylacetate	74.54 (50.14~145.18)b	0.67 ± 0.12	21.71	0.95
Citronellyl acetate	104.08 (90.93~121.68)b	0.48 ± 0.03	4.62	0.88
β-Caryophyllene	208.77 (191.42~229.57)a	0.24 ± 0.01	1.445	0.86

LC_50_ values with non-overlapping 95% confidence intervals (CIs) indicate significant differences between compounds (P< 0.05). Different letters follow LC_50_ value mean significantly different.

### 3.3 Vapor toxicity to dust mites

The fumigation efficacy of both the essential oil and its seven principal compounds was evaluated through vapor-phase bioassays conducted at twice the concentration of their respective LC_50_ values. Mortality rates were analyzed to determine fumigation effectiveness, where a positive correlation was observed between mortality rate and increased toxicity levels across treatments within closed versus open groups (P< 0.0001; **Tables 5**, **6**). Both citronellal and ethyl phenylacetate achieved complete mortality against both *Dermatophagoides* species under closed conditions, even at the two lowest tested concentrations (127.88 μL/cm² for citronellal and 145.65 μL/cm² for ethyl phenylacetate, **Table 5**), highlighting their higher efficacy; additionally, the mortality effects associated with essential oils, 1,8-cineole, and citronellyl acetate exceeded 65% in closed containers (**Tables 5**, **6**). This underscores the potential utility of citronellal, ethyl phenylacetate, essential oils, 1,8-cineole, and citronellyl acetate for controlling dust mites via fumigation.

**Table 5:** Fumigant toxicity of *Eucalyptus citriodora* oil and the seven main compounds to adult *Dermatophagoides pteronyssinus* using a vapor-phase mortality bioassay after 24 h exposure.

Compounds	Concentration(μL/cm2)	(Mortality ± SE)%		P-value
		Vapor in closed container	Vapor in open container	
Essential oil	169.06	65 ± 6.91	22.78 ± 4.91	<0.0001
1,8-Cineole	152.81	78.89 ± 6.89	14.44 ± 3.44	<0.0001
Citronellol	228.05	47.22 ± 5.74	12.22 ± 5.02	<0.0001
Citronellal	127.88	100	13.33 ± 2.98	<0.0001
(-)-Globulol	303.58	36.67 ± 5.96	8.33 ± 1.83	<0.0001
Ethyl phenylacetate	145.65	100	15.56 ± 4.55	<0.0001
Citronellyl acetate	191.64	76.67 ± 4.71	21.11 ± 3.44	<0.0001
β-Caryophyllene	409.67	16.67 ± 2.98	2.78 ± 2.51	<0.0001

**Table 6:** Fumigant toxicity of *Eucalyptus citriodora* oil and the seven main compounds to adult *Dermatophagoides farinae* using a vapour-phase mortality bioassay after 24 h exposure.

Compounds	Concentration(μL/cm^2^)	(Mortality ± SE)%		P-value
		Vapor in closed container	Vapor in open container	
Essential oil	151.77	88.89 ± 4.55	27.22 ± 5.74	<0.0001
1,8-Cineole	142.27	95.56 ± 5.02	23.33 ± 4.71	<0.0001
Citronellol	226.3	48.33 ± 7.53	15 ± 3.5	<0.0001
Citronellal	121.43	100	11.11 ± 3.44	<0.0001
(-)-Globulol	282.09	37.78 ± 4.55	8.33 ± 3.5	<0.0001
Ethyl phenylacetate	149.08	100	11.11 ± 3.44	<0.0001
Citronellyl acetate	208.16	70 ± 5.58	15 ± 4.59	<0.0001
β-Caryophyllene	417.54	17.22 ± 3.9	3.33 ± 2.11	<0.0001

### 3.4 Repellent effect

**Figure 2:**
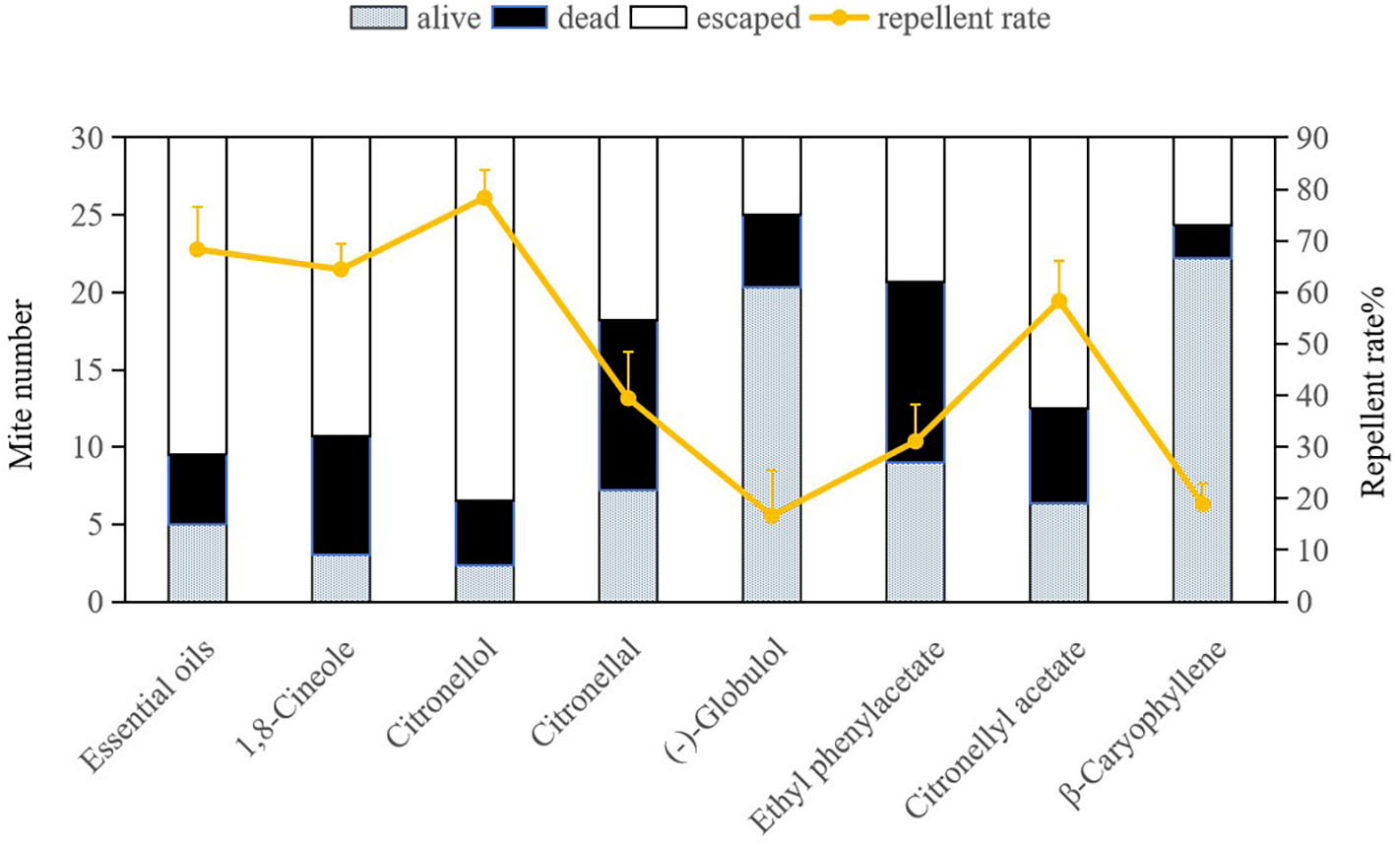
Repellent effects of *Eucalyptus citriodora* essential oil and its seven main compounds on *Dermatophagoides pteronyssinus.* Escaped means the mites were repelled from treat quadrant to control quadrant in the same patric dish. Data were analyzed using ANOVA followed by Bonferroni *post-hoc* tests; bars with different letters indicate significant differences (P< 0.05).

**Figure 3:**
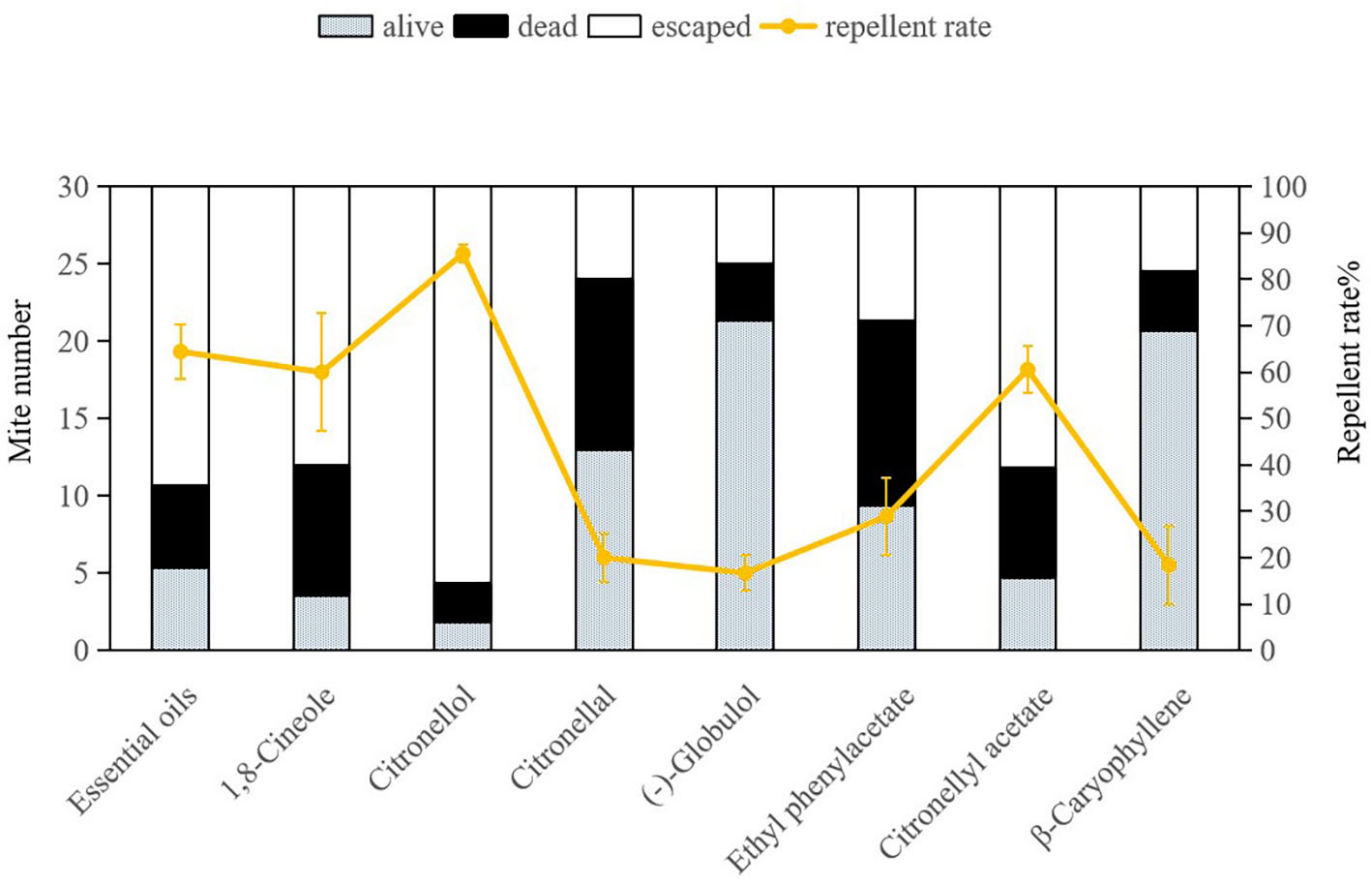
Repellent effects of *Eucalyptus citriodora* essential oil and its seven main compounds on *Dermatophagoides farinae.* Data were analyzed using ANOVA followed by Bonferroni *post-hoc* tests; bars with different letters indicate significant differences (P< 0.05).

The repellent efficacy of *E. citriodora* essential oil along with its seven key compounds against dust mites is illustrated in **Figure 2** and **Figure 3**. Due to the fumigant toxicity as well as the low concentration (LC_50_) of the essential oil and chemical compounds, both dead and alive (non escaping) mite data were considered when calculating the repellent rate. Citronellol demonstrated the highest repellent rate against both dust mite species *D. pteronyssinus* and *D. farinae* (78.33% and 85.56%, respectively), and significantly surpassing other tested compounds (P< 0.0001). The repellent rates of essential oil, 1, 8-cineole, and citronellyl acetate against both dust mite species exceeded 50%, which were also significantly higher than those of the remaining four compounds (P< 0.0001). Repellency rates were analyzed via ANOVA following arcsine square root transformation of mortality percentages to meet normality assumptions. Significant differences among compounds were identified using the Bonferroni multiple-comparison procedure (P< 0.001), with citronellol exhibiting the highest repellency (78.33-85.56%) and statistically distinct from all other tested substances. This indicates that citronellol, 1,8-cineole, and citronellyl acetate play crucial roles in the overall repellency effects of *E. citriodora* essential oil against both dust mite species. In our study, there was no indication that dead dust mites repelled living mites.

## 4 Discussion

The present study aimed to evaluate the toxicity and repellent effects of *E. citriodora* essential oil and its main compounds against two common dust mite species, *D. pteronyssinus* and *D. farinae*. The results provide valuable insights into the potential use of this essential oil as a natural and environmentally friendly dust mite control agent.

The GC-MS analysis revealed that seven chemicals (1,8-cineole, citronellol, citronellal, (-)-globulol, citronellyl acetate, β-caryophyllene, and ethyl phenylacetate) were the main compounds of *E. citriodora* essential oil. Among these, citronellol, citronellal and citronellyl acetate were the predominant constituents in the volatile fraction. Citronellol and citronellal have been previously reported to exhibit insecticidal and repellent activities against various arthropod pests, including *Aedes aegypti* (Patel et al., 2023), *Myzus persicae* (Ahmed et al., 2022), and *Musca domestica* (Ahmadi et al., 2022; Pavela et al., 2022), which aligns with their demonstrated efficacy against dust mites in our study. Notably, our findings contrast and complement recent research on other botanical acaricides, *Borneol essential* oil (BEO) achieves >95% repellency at 0.5 mg/ml (Yu et al., 2022), comparable to the 85.56% repellency of citronellol in our study. Additionally, *Abies sachalinensis* oil inhibits dust mite allergen Der f 2 by reducing protein abundance (Lin et al., 2022), whereas *E. citriodora* acts directly via toxicity and repellency.

The contact and fumigant toxicity bioassays demonstrated that *E. citriodora* essential oil and several of its compounds exhibited significant toxicity against both dust mite species. Notably, citronellal, 1,8-cineole, and ethyl phenylacetate showed the lowest LC_50_ values, indicating the highest toxicity among the tested compounds. Meanwhile, the essential oil itself also showed strong toxicity, with an LC_50_ value below 85 μL/cm^2^ for both mite species, similar toxicity to above three singular constituents. It means that these three components of the *E. citriodora* essential oil (citronellal, 1,8-cineole, and ethyl phenylacetate) may be the key factors that enable the essential oil to kill the dust mites. This finding is in line with previous studies that have reported potent insecticidal activity of citronellal, ethyl phenylacetate, and 1,8-cineole (Sabio et al., 2024; Yan et al., 2024). The vapor-phase mortality bioassay further confirmed the biotoxicity of the essential oil and its compounds, with citronellal and ethyl phenylacetate showing 100% mortality in closed containers. This indicates that these compounds could effectively be used to control dust mites through vapor action alone, which is known to be a desirable property for a pest control agent, as it does not require direct contact with the target organism. Future studies should investigate that slow-release formulations of high-volatility compounds would be the important practice of dust mite IPM.

The repellent bioassay revealed that citronellol exhibited the highest repellent efficacy against *D. pteronyssinus* and *D. farinae*, with mean repellency rates of 85.56 ± 2.14% and 78.33 ± 1.89% at 60 min post-treatment, respectively. These values were significantly higher than those of other tested compounds. These findings indicated that citronellol is likely the principal contributor to the repellency of *E. citriodora* essential oil. The essential oil, 1,8-cineole, and citronellyl acetate also showed significant repellent effects, with repellency rates above 50%. Previous study also demonstrated that citronellol, 1,8-cineole, and citronellyl acetate repelled insects and mites (Han et al., 2011; Deletre et al., 2022; Ayllón-Gutiérrez et al., 2023; Kaneko et al., 2024; Kunnathattil et al., 2024; Yan et al., 2024).

The efficacy of citronellol and citronellal is closely linked to their physicochemical properties. Citronellal’s low molecular weight (154.23 g/mol) and high vapor pressure (0.85 hPa at 25°C) enable rapid vapor-phase diffusion, explaining its 100% mortality in closed-container assays even at low concentrations (127.88 μL/cm² for *D. pteronyssinus*). In contrast, β-caryophyllene’s larger molecular size (204.32 g/mol) and negligible volatility (0.045 hPa) correlate with its poor fumigant efficacy (<20% mortality), illustrating the critical role of vapor pressure in gaseous toxicity. Citronellol, despite lower volatility (0.02 hPa), exhibits strong repellency (85.56% against *D. farinae*), likely due to its alcohol functional group interacting with mite chemoreceptors, as hypothesized in insect repellency studies (Dawood and Snyder, 2020). This aligns with its structural similarity to known arthropod repellents, where hydroxyl moieties enhance binding to olfactory receptors (Zhang et al., 2019).

The findings of this study suggest that *E. citriodora* essential oil and its main compounds, particularly citronellal and citronellol, could serve as effective dust mite control bioagents. Their natural origin, strong toxicity, and repellent effects make them promising alternatives to traditional chemical control methods, which can have negative impacts on human health and the environment. To translate these findings into practical use, future work should explore slow-release formulations for citronellal/ethyl phenylacetate and fabric treatments with citronellol, plus optimize concentrations to balance efficacy and safety. Field testing of these methods in homes will also confirm real-world mite control and support household application.

## 5 Conclusions

This study demonstrates that *Eucalyptus citriodora* essential oil and its key components, particularly citronellal and citronellol, exhibit notable biotoxicity and repellent activity against *Dermatophagoides pteronyssinus* and *D. farinae*. Gas chromatography-mass spectrometry identified seven major compounds, with citronellal, citronellol, and citronellyl acetate dominating the volatile fraction. Contact and fumigant toxicity assays revealed that the essential oil and compounds like citronellal, 1,8-cineole, and ethyl phenylacetate primarily exert acaricidal effects via contact and fumigant actions while citronellol drives repellency likely through interactions with mite chemoreceptors. These findings underscore the potential of *E. citriodora* essential oil as a natural alternative to synthetic pesticides for dust mite control, while the proposed strategies provide actionable guidance for translating laboratory results into scalable indoor pest management solutions. Future research should focus on optimizing formulation stability, validating field efficacy in residential settings, and defining safety thresholds to support regulatory approval.

## Data Availability

The datasets presented in this study can be found in online repositories. The names of the repository/repositories and accession number(s) can be found below: https://doi.org/10.6084/m9.figshare.28632437.v1.
